# Engineering Immunomodulatory Biomaterials for Regenerating the Infarcted Myocardium

**DOI:** 10.3389/fbioe.2020.00292

**Published:** 2020-04-07

**Authors:** Nora Bloise, Isobel Rountree, Collin Polucha, Giulia Montagna, Livia Visai, Kareen L. K. Coulombe, Fabiola Munarin

**Affiliations:** ^1^Department of Molecular Medicine, Center for Health Technologies (CHT), INSTM UdR of Pavia, University of Pavia, Pavia, Italy; ^2^Department of Occupational Medicine, Toxicology and Environmental Risks, ICS Maugeri, IRCCS, Pavia, Italy; ^3^Center for Biomedical Engineering, School of Engineering, Brown University, Providence, RI, United States; ^4^Department of Electrical, Computer and Biomedical Engineering, Centre for Health Technologies (CHT), University of Pavia, Pavia, Italy

**Keywords:** immune engineering, biomaterials, angiogenesis, revascularization, myocardial infarct, macrophages, cytokines

## Abstract

Coronary artery disease is a severe ischemic condition characterized by the reduction of blood flow in the arteries of the heart that results in the dysfunction and death of cardiac tissue. Despite research over several decades on how to reduce long-term complications and promote angiogenesis in the infarct, the medical field has yet to define effective treatments for inducing revascularization in the ischemic tissue. With this work, we have developed functional biomaterials for the controlled release of immunomodulatory cytokines to direct immune cell fate for controlling wound healing in the ischemic myocardium. The reparative effects of colony-stimulating factor (CSF-1), and anti-inflammatory interleukins 4/6/13 (IL4/6/13) have been evaluated *in vitro* and in a predictive *in vivo* model of ischemia (the skin flap model) to optimize a new immunomodulatory biomaterial that we use for treating infarcted rat hearts. Alginate hydrogels have been produced by internal gelation with calcium carbonate (CaCO_3_) as carriers for the immunomodulatory cues, and their stability, degradation, rheological properties and release kinetics have been evaluated *in vitro*. CD14 positive human peripheral blood monocytes treated with the immunomodulatory biomaterials show polarization into pro-healing macrophage phenotypes. Unloaded and CSF-1/IL4 loaded alginate gel formulations have been implanted in skin flap ischemic wounds to test the safety and efficacy of the delivery system *in vivo*. Faster wound healing is observed with the new therapeutic treatment, compared to the wounds treated with the unloaded controls at day 14. The optimized therapy has been evaluated in a rat model of myocardial infarct (ischemia/reperfusion). Macrophage polarization toward healing phenotypes and global cardiac function measured with echocardiography and immunohistochemistry at 4 and 15 days demonstrate the therapeutic potential of the proposed immunomodulatory treatment in a clinically relevant infarct model.

## Introduction

Ischemic heart disease, or myocardial infarction (MI), is the leading cause of death globally, causing 1.8 million deaths per year in Europe (40% of all deaths) and 647000 deaths per year (25% of all deaths) in the United States (Wilkins et al., 2017; Benjamin et al., 2019). MI is typically caused by the formation of atherosclerotic plaques that occlude a coronary artery, resulting in cardiomyocyte death due to insufficient oxygen perfusion (Shimokawa and Yasuda, 2008; Frank et al., 2012). Despite decades of research, treatments for improving vascularization of the infarcted myocardium are still needed, and bioengineering approaches are starting to emerge (Kellar et al., 2005; Dvir et al., 2009; Fleischer et al., 2017). Recent studies have demonstrated the crucial role of reparative macrophage phenotypes for initiating angiogenesis and reparative processes in the heart and in other tissues of the body (Godwin et al., 2013; Aurora et al., 2014; Cattin et al., 2015). Cardiomyocyte death following an ischemic injury triggers the innate immune response, and a plethora of immune cells, including leukocytes, monocytes and macrophages, are rapidly recruited and activated in the injured site (Sica and Mantovani, 2012). In this early inflammatory phase, monocytes are polarized into pro-inflammatory subtypes that secrete inflammatory chemokines and are responsible for the phagocytosis of apoptotic cells and debris. After the acute inflammatory phase, a subpopulation of reparative macrophages initiates the healing process by secreting pro-angiogenic growth factors such as vascular endothelial growth factor-α (VEGF-α), platelet-derived growth factor (PDGF), transforming growth factor-β (TGF-β) and insulin-like growth factor-1 (IGF-1) (Sica and Mantovani, 2012; Butoi et al., 2016; Hesketh et al., 2017) to stimulate endothelial cell morphogenesis and activation (Kalucka et al., 2017). Macrophages are extremely diverse and plastic cells, and their phenotypic subset is determined by environmental signals (Murray, 2017). Beyond the pro-inflammatory and pro-healing subtypes, there is a continuum of functional states, with some macrophages displaying characteristics of both classically defined phenotypes (Mosser and Edwards, 2008). In the infarcted myocardium, the controlled recruitment and activation of monocytes and macrophages is required for orchestrating tissue repair and neoangiogenesis to limit excessive scarring and fibrosis (Frangogiannis, 2018; Swirski and Nahrendorf, 2018). Unbalanced or impaired activation of the cells of the immune system after myocardial ischemia results in maladaptive ventricular dilatation, systolic or diastolic dysfunction and myocardial stiffness (Frangogiannis, 2014).

With this study, we aim at exploiting immune-mediated mechanisms for stimulating wound healing in the ischemic myocardium by use of biomaterials designed for the controlled release of selected immunomodulatory cytokines. Colony-stimulating factor (CSF-1) and interleukin 4 (IL4) have been widely used to elicit human blood monocyte polarization into reparative macrophage phenotypes (Rovida et al., 2001; Boulakirba et al., 2018) and to modulate endothelial cell function (Bussolino et al., 1991). However, a potential involvement of CSF-1 and IL4 in atherosclerosis and other chronic inflammatory diseases has been identified (Smith et al., 1995; Qiao et al., 1997; von der Thusen et al., 2003; Shaposhnik et al., 2010), though the mechanisms by which these cytokines contribute to the development of these conditions remain unclear. In order to minimize risks of atherosclerotic activation of circulating CSF-1 and IL4 *in vivo*, a controlled and localized delivery of these factors after MI is necessary. Here we have designed an alginate-based biomaterial system for the release CSF-1 and IL4 to accelerate the polarization of blood monocytes into pro-healing macrophage phenotypes. Our hypothesis is that the activation of immune-mediated reparative mechanisms will promote wound healing and limit myocardial dysfunction after the infarct. We assess the ability of this new immunomodulatory treatment to revascularize ischemic wounds and to repair damaged tissues with clinically relevant predictive studies *in vitro* with use of human peripheral blood monocytes and *in vivo* with rodent models of ischemic skin flap and myocardial infarction.

## Materials and Methods

### Isolation of Human Monocytes and Cell Culture

Peripheral blood was obtained from healthy donors (Centro Lavorazione e Validazione, Servizio di Immunoematologia e Medicina Trasfusionale, Fondazione I.R.C.C.S. Policlinico San Matteo, Pavia, Italy) according the following Italian Normative References: Decreto Ministero della Salute 2 novembre 2015 n.69 “Disposizioni relative ai requisiti di qualità e sicurezza del sangue e degli emocomponenti” and Accordo Stato-Regioni n.225/CSR 13 dicembre 2018 “Schema-tipo di convenzione per la cessione del sangue e dei suoi prodotti per uso di laboratorio e per la produzione di dispositivi medico-diagnostici *in vitro*.” Written informed consent was obtained from all the participants enrolled in this study. Peripheral blood mononuclear cells (PBMC) were isolated from buffy coat by density gradient centrifugation using Histopaque^®^-1077 (1.077 g ml^–1^; Sigma-Aldrich, St. Louis, MO, United States) as previously described (Kleiveland, 2015). Human CD14 + monocytes were then obtained by Monocyte Isolation Kit II (Miltenyi Biotec, Bergisch Gladbach, Germany) according to the manufacturer instructions. After isolation, CD14 + monocytes were counted by Trypan Blue assay (Sigma-Aldrich) and were plated in 24 well plates at a density of 1 × 10^5^ cells/well. All cells were cultured in RPMI 1640 medium supplemented with 2 mM/L L-glutamine (Life Technologies, Frederick, MD), 10% heat inactivated FBS (Sigma-Aldrich), 100 U/ml penicillin (Life Technologies), and 100 mg/ml streptomycin (Life Technologies) at 37°C in a humidified incubator with 5% CO_2_.

### Production and Characterization of Alginate Hydrogels

#### Production of Alginate Hydrogels

For the production of unloaded alginate hydrogels, alginate (Sigma, 180947), D-(+)-gluconic acid δ-lactone (GDL, Sigma, G4750) and calcium carbonate (Fisher Scientific, S25220A and 10617581) were used as received. Alginate hydrogels were produced from aqueous alginate solutions (2–6% w/v) mixed with 10 mg/mL GDL. Calcium carbonate (2.5–40 mg/mL) was suspended with distilled water and sonicated at room temperature for 10 min. The calcium carbonate suspension was then added to the alginate – GDL solution to produce different hydrogel formulations, named AC2.5 (Alginate 1%, CaCO_3_ 2.5 mg/mL), AC5 (Alginate 1%, CaCO_3_ 5 mg/mL), and AC10 (Alginate 1%, CaCO_3_ 10 mg/mL). The gelling mixture was placed in 24 well plates (0.5 mL/well) and was left to gel at *T* = 37°C for 10–60 min. Gelling time was determined with the test-tube inversion assay, by tilting the plates containing the polymeric mixture every 2 min, until no flow was observed.

#### Swelling and Degradation Studies

Swelling and degradation studies were performed on hydrogel samples cut with a 6 mm biopsy punch and incubated in PBS at 37°C up to 14 days, by measuring wet and dry weight at each time point (*t* = 0, 1, 3, 7, and 14 days) to determine the% wet and dry weight variation (ΔW_*w*_ and ΔW_*d*_), as previously described in Moreira et al. (2014).

### Rheological Properties of Alginate Hydrogels

To evaluate the viscoelastic properties of alginate hydrogels, shear (0.01–100 s^–1^) and frequency (10–300 rad/s) sweep tests were conducted on AC5 and AC10 samples at *T* = 23°C by use of a rheometer (TA Instruments) equipped with an 8 mm plate-plate geometry.

#### Evaluation of Alginate Hydrogel Cytotoxicity

AC5 and AC10 hydrogel samples were incubated in RPMI medium (Life Technologies) at 37°C, 5% CO_2_ up to 10 days. At the end of the incubation period, the supernatant (containing the gel degradation products) was collected and added (undiluted or diluted 1:2 and 1:4 in complete culture medium) to isolated human CD14 + monocyte cultures, previously seeded at a density of 1 × 10^5^ cells per well in 24-well plates. After 24 h, cell viability was assessed by the quantitative 3-[4,5-dimethylthiazol-2-yl]-2,5-diphenyl tetrazolium bromide (MTT) test (Sigma-Aldrich) as previously described (Gualandi et al., 2016).

### Release Profiles of Bovine Serum Albumin (BSA) From Alginate Hydrogels

The release kinetics of alginate hydrogels were evaluated by loading the biomaterials with different amounts of a model protein, bovine serum albumin (BSA, molecular weight = 66 kDa; Sigma-Aldrich). The model protein was added to AC5 and AC10 gelling mixtures in the following concentrations: 0, 50, 100, and 150 μg/mL. After gelation, loaded and unloaded hydrogels were submersed in 1 mL of 1× PBS (pH 7.4) at 37°C. At selected time points (1–10 days) the incubation solution was completely collected and 1 mL of fresh PBS was added to maintain perfect sink conditions. The concentration of proteins in the supernatants was determined using the Bicinchoninic Protein Assay Kit (BCA assay, EuroClone S.p.A), according to the manufacturer protocol. Standard BSA curves were used to determine the protein concentrations in each sample.

Fluorescein isothiocyanate-labeled bovine serum albumin (FITC-BSA, Sigma-Aldrich) was immobilized in the hydrogels in order to evaluate the homogeneity of distribution of the protein by visual observation using a confocal laser scanning microscope (CLSM) model TCS SP8 (Leica Microsystems, Bensheim, Germany, Oil 40) at 24 h from loading.

### Evaluation of the Immunomodulatory Effects of Colony- Stimulating Factor 1 and Interleukins 4/6/13 on Human Monocytes Isolated From Peripheral Blood

We have loaded AC10 hydrogels (0.5 mL final volume) with: (i) no cytokines (unloaded control); (ii) CSF-1 (150 ng/mL); (iii) CSF-1 (150 ng/mL) and IL4 (150 ng/mL, GenScript Corporation); and (iv) CSF-1 (150 ng/mL) with a combination of IL4, IL6, and IL13 (each cytokine at a concentration of 150 ng/mL, GenScript Corporation). Hydrogels were then maintained in monocyte culture medium for 10 days. Then, purified monocytes (1 × 10^5^ cells seeded per well in 24-well) were incubated in the presence of the hydrogel extracts (1 mL) for 10 days. As control, human CD14 + monocytes were exposed for the same time to medium containing freshly prepared differentiation factors (20 ng/mL, alone or in combination), according to literature protocols (Zarif et al., 2016; Supplementary Figure S2).

Macrophage polarization toward pro-healing phenotypes was assessed after 10 days of culture by optical light microscopy (for morphology evaluation) and by immunofluorescence with specific antibodies for the expression of the haptoglobin–hemoglobin receptor CD163 and the mannose receptor CD206, strongly expressed on the reparative M2c and M2a macrophage subtypes, respectively (Yang et al., 2014), as described in the Supplementary Material. Quantification of CD163 or CD206 positive cells was determined by acquiring images with a confocal laser scanning microscope (TCS SP8, Leica Microsystems, Bensheim, Germany, Oil 40× and 63×), and processing them with ImageJ-Analyzer software (Schneider et al., 2012; van den Bosch et al., 2017). The number of CD163 + and CD206 + cells was expressed as total number of positive cells/total number of nuclei, analyzing five fields of view per sample.

### Wound Healing Assessment in the Ischemic Skin Flap Model

The ischemic skin flap surgical procedure has been conducted according to the United States NIH Policy on Humane Care and Use of Laboratory Animals and the Brown University and Rhode Island Hospital Institutional Animal Care and Use Committee (IACUC protocol No. 1702000256). Male Sprague Dawley wild type rats, strain 001 (Charles Rivers), with an average weight of 310 ± 61 g, were sedated with 2.5–3% isoflurane, the skin of the back was shaved and four full-thickness excisional wounds were created with a 6 mm biopsy punch in the designated ischemic area. A bipedicled 4 cm × 6 cm skin flap was created, dissecting the panniculus carnosus muscle from the wound bed. A sterile 4 cm × 6 cm silicone sheet was inserted beneath the flap, to prevent perfusion of the flap from the underlying tissue. The flap incision was closed using surgical skin glue. A non-ischemic wound was created lateral to the bipedicled flap and used as non-ischemic control. Each animal received the following treatments in the four ischemic wounds: (i) no treatment, (ii) unloaded AC10, and (iii) AC10 loaded with 2 μg CSF-1 and 2 μg IL4. Alginate hydrogels (*d* = 6 mm) were inserted in the ischemic wounds and maintained in the defect with two 4-0 nylon loop sutures. A transparent film dressing was applied to keep the wound environment clean. Rats recovered with saline and analgesic (Buprenorphine SR, 1 mg/kg). Digital images of the ischemic and non-ischemic wounds were acquired up to 21 days, after light sedation of the animals (1.5% isoflurane). Measurement of the epithelial gap was conducted using Fiji software. Wound tissues were harvested post-euthanasia making a square- shaped incision around the wound to include some healthy tissue. Samples were fixed in 4% paraformaldehyde (PFA) overnight and processed for subsequent paraffin embedding. Histological analysis was conducted with hematoxylin/eosin staining, RECA-1 and CD68 for phenotypical assessment of the cell population present in the ischemic wounds (staining protocols are described in detail in the Supplementary Material).

### Injection of the Immunomodulatory Biomaterials in a Rodent Model of Infarcted Myocardium

All experiments were performed according to United States. NIH Policy on Humane Care and Use of Laboratory Animals and the Brown University and Rhode Island Hospital Institutional Animal Care and Use Committee (IACUC protocol No. 1702000256). Ischemia/reperfusion surgery was performed on Sprague Dawley male rats, strain 001 (Charles Rivers), 317 ± 37 g of weight. Animals were anesthetized with intraperitoneal injection of ketamine (80–100 mg/kg) and xylazine (5–10 mg/kg), were intubated and ventilated (1.8–2.5 mL/cycle, 65–90 cycles/min) with oxygen. After opening the chest cavity and exposing the heart, the left anterior descending coronary artery was ligated with a 7–0 polypropylene suture for 1 h, followed by reperfusion and immediate injection of the therapeutics. Three types of injections were performed: (i) injectable saline, (ii) unloaded AC10, and (iii) CSF-1 (2 μg)- and IL4 (2 μg)- loaded AC10. For each animal, three injections were performed in three different regions of the infarct (apical, mid anterior and mid anterolateral). Echocardiographic measurements performed at 4 days post MI allowed for evaluation of the left ventricular function. Animals presenting small infarcts, with fractional shortening above 45%, were excluded from this study. Echocardiography was performed (using GE Vivid 7 with a 10S 4–10 MHz transducer probe) under light inhaled isoflurane (1–2%) prior to the MI (baseline) and at 4 and 15 days post MI. Measurement of dimensional parameters (cavity dimensions and wall thickness) was conducted in M-mode, and fractional shortening and ejection fraction were calculated.

After euthanasia, hearts were harvested and post-fixed in 4% PFA over night at 4°C. Hearts were then cut by use of a coronal heart matrix to obtain 2 cm-thick cross-sectional slices and put in paraffin blocks subsequently cut in 5 μm-thick sections. Picrosirius red/fast green, cardiac troponin T (cTnT) and CD68 staining were performed to assess infarct area cardiomyocytes and macrophages density in the injured site Immunohistochemistry with CD68/CD206/DAPI and CD68/CD80/DAPI allowed to detect specific macrophages subtypes in the infarct and border zone (staining protocols are described in the Supplementary Material).

Infarct size was evaluated at 4 and 15 days post MI using Fiji software. Infarct area was measured in picrosirius red/fast green stained heart slices by manual segmentation of the red-stained collagenous scar tissue. The infarct area was then normalized to the left ventricle area. At least two slices were analyzed per each heart at the apical to mid-papillary level (s1–s3) and *n* = 3 hearts were analyzed per each group.

### Statistical Analysis

Statistical analysis was carried out using GraphPad Prism 8.0 (GraphPad Inc., San Diego, CA, United States). Analysis was performed using one-way and two-ways analysis of variance (ANOVA), followed by Bonferroni or Tukey *post hoc* test (significance level of 0.05).

## Results

### Alginate Hydrogels Are Suitable Soft and Biodegradable Biomaterials for Cytokine Delivery

**FIGURE 1:**
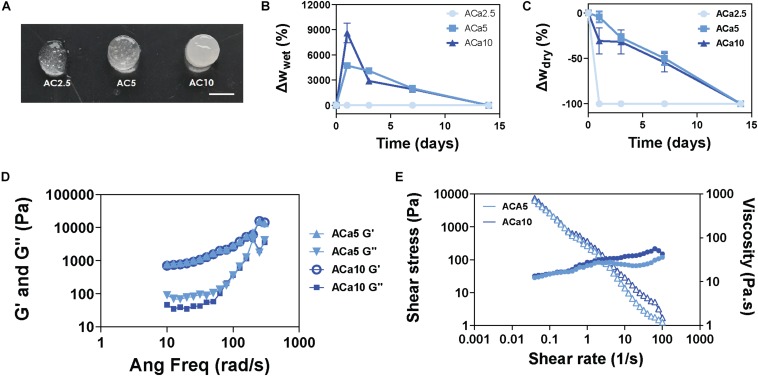
Soft and biodegradable alginate hydrogels are produced by internal crosslinking with calcium carbonate. **(A)** Images of AC2.5, AC5, and AC10 hydrogels, scale bar = 5 mm. **(B)** Wet weight variation (swelling) of hydrogels compared to their initial weight at day 0. **(C)** Dry weight variation (mass loss) of hydrogels compared to the initial dry weight at day 0. **(D)** Rheological characterization of storage (G′) and loss (G″) moduli of AC5 and AC10 by frequency sweep test at 0.5% strain and *T* = 23°C and **(E)** shear rate dependence of shear stress (filled circles) and viscosity (open triangles) at *T* = 23°C.

Soft alginate hydrogels have been produced by internal gelation, using calcium carbonate and glucono-δ-lactone for lowering the pH of the gelling mixture, allowing slow dissociation of calcium ions and homogeneous gel formation. Varying the final concentration of alginate (in the range of 1-4% w/v) and CaCO_3_ (in the range of 2.5–20 mg/mL), we were able to produce hydrogels characterized by different gelling time and rheological properties. Amongst the several formulations produced, alginate 1% - CaCO_3_ 2.5 mg/mL (AC2.5), alginate 1% - CaCO_3_ 5 mg/mL (AC5) and alginate 1% - CaCO_3_ 10 mg/mL (AC10) exhibited a gelling time of 5–10 min at *T* = 37°C, ideal for the production of *in situ*-gelling injectable biomaterials (Figure 1A). Swelling/stability studies performed on AC2.5, AC5, and AC10 showed complete degradation of AC2.5 hydrogels in 1 day, while AC5 and AC10 formulations degraded in 14 days (Figures 1B,C). Rheological characterization of AC5 and AC10 hydrogels was performed within the linear viscoelastic region, determined with a strain sweep test ranging from 0.1 to 1%, at an oscillatory frequency of 10 rad/s. Storage (G′, Pa) and loss (G″, Pa) moduli, measured with a frequency sweep test at 0.5% strain, showed negligible differences between the AC5 and AC10 hydrogels (Figure 1D) and typical gel-like behavior, characterized by the predominance of the solid-like component (G′) over the viscous component (G^″^). Furthermore, AC5 and AC10 hydrogels exhibited a shear thinning behavior, manifested as a slight increase of shear stress (circles) and decrease of viscosity (triangles) at increasing shear rate at *T* = 23°C (Figure 1E). Comparing the results obtained in this work at *T* = 23°C with the results of other studies performed on alginate hydrogels at *T* = 37°C (Duan et al., 2017), we expect no or modest decrease in the viscosity and elastic modulus at physiological temperature.

Cell viability test by the colorimetric MTT (3-(4,5-dimethylthiazol-2-yl)-2,5-diphenyltetrazolium bromide) assay, detecting cell viability and metabolic activity, showed that the degradation products of hydrogels did not induce toxic effects in human CD14 + monocytes cultures. Indeed, after 24 h of co-incubation with AC5 or AC10 extracts, the average cell viability was in the 90–94% range, with no statistical difference in cell viability (*p* > 0.05) between all tested samples and in comparison with cells cultured in degradation products free-RPMI medium.

### Immobilization of a Model Protein in Alginate Hydrogels Enables Controlled and Localized Release

**FIGURE 2:**
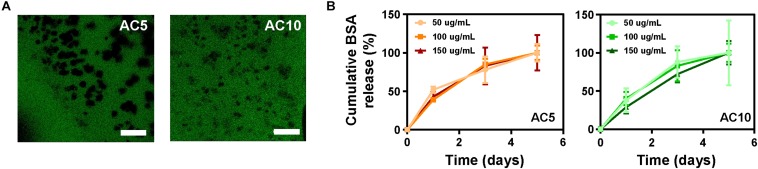
BSA is released from AC5 and AC10 in 5 days. **(A)** Representative confocal laser scanning microscopy images of AC5 (left) and AC10 (right) hydrogels loaded with BSA-FITC protein acquired after 24 h in phosphate buffered saline (PBS). Scale bar: 100 μm. **(B)** Quantification of BSA content in PBS supernatant collected from hydrogels at different incubation times. Data are represented as cumulative percentage release relative to the initial loading dose ± SD (*n* = 3).

A model protein (bovine serum albumin, BSA) was immobilized at different concentrations (50–150 μg/mL) in AC5 and AC10 hydrogels to predict the release kinetics of the immunomodulatory cytokines. Images of the hydrogels acquired at the confocal microscope showed homogeneous dispersion of FITC-labeled BSA in the hydrogels (Figures 2A,B). The release profile of BSA from alginate hydrogels, measured with the BCA assay, a colorimetric assay for quantitation of protein release, showed a gradual release of the protein over a period of 5 days. No statistical differences were detected when comparing the release of increasing concentrations of BSA at each time point from AC5 and AC10 hydrogels (Figures 2A,B).

### Colony- Stimulating Factor 1 and Interleukins 4, 6, and13 Released From Alginate Hydrogels Stimulate Polarization of Human Monocytes Isolated From Peripheral Blood

Based on evidence in the literature (Mia et al., 2014; Holloway, 2018), we have induced polarization of CD14 + human monocytes (purified from peripheral blood and characterized by flow cytometry analysis, Supplementary Figure S1) into pro-healing macrophage phenotypes (the so-called M2 macrophages) by adding 20 ng/mL CSF-1 for 6–13 days directly to the culture medium.

Addition of anti-inflammatory interleukins (IL4, 20 ng/mL or a combination of IL4/IL6/IL13, 20 ng/mL each) notably improved the yield of polarization of monocytes toward reparative macrophage phenotypes, as confirmed by the increasing expression of CD163 and CD206 markers, associated to the M2c and M2a reparative macrophage phenotypes (Supplementary Figure S2).

**FIGURE 3:**
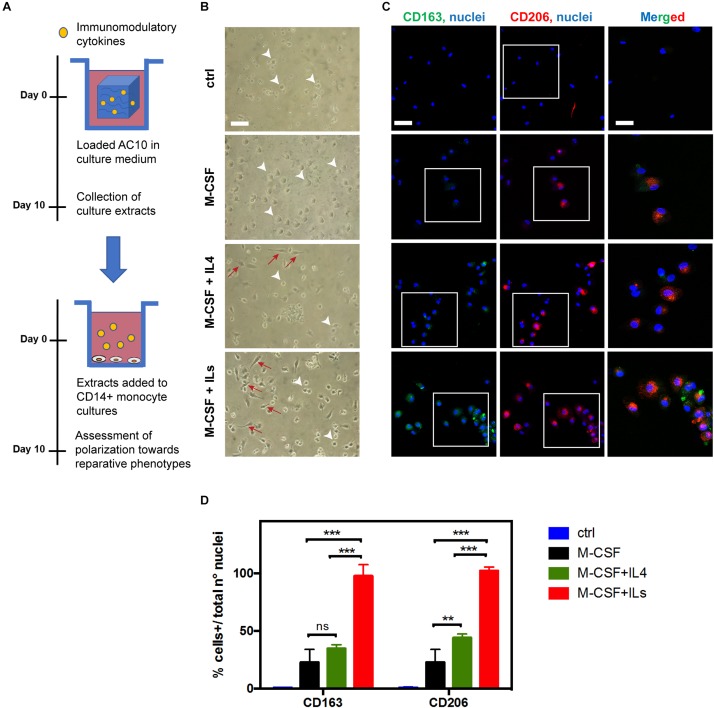
Optimized combinations of immunomodulatory cytokines released from AC10 hydrogels promote monocytes polarization toward healing macrophage phenotypes. **(A)** Isolated human CD14 + monocytes were cultured for 10 days in the presence of medium containing cytokines obtained from solid hydrogels after 10 days of incubation. **(B)** Representative optical light microscopy showing inflammatory macrophages phenotypes characterized by the round morphology (white arrowheads) and anti-inflammatory macrophage subsets with elongated morphology (red arrows). Scale bar = 50 μm. **(C)** Confocal images obtained from the specified conditions. Expression of M2-macrophage markers, CD163 (green) and CD206 (red) was evaluated. Scale bar = 50 μm. Nuclei were counterstained with Hoechst 33342 (blue). Rectangles in panel **(C)** represent cells in magnified area, where merged CD163/CD206/nuclei signals are shown (Scale bar = 20 μm). **(D)** Number of CD163 + or CD206 + cells was expressed as total positive cell per total nuclei number per analyzed field (ns = *p* > 0.05, **p < 0.01; ***p < 0.001, *n* = 3).

The bioactivity and efficacy of the immune-modulating cytokines released from AC10 hydrogels were investigated by evaluating morphology and the expression of pro-healing macrophage markers expressed by treated monocytes by optical light microscopy and immunohistochemistry, respectively (Figure 3A). As showed in Figure 3B, CSF-1 extracts from AC10 induced the differentiation of reparative macrophage phenotypes in 10 days, with similar effectiveness compared to the addition of fresh cytokines to the culture medium (Supplementary Figure S2A). Striking differences were noted among the different media eluting from hydrogels groups. The percentage of small and rounded cells, associated with pro-inflammatory macrophage subsets, was significantly higher in cultures of untreated monocytes and monocytes treated with CSF-1 alone, compared to the more elongated morphology observed for cells cultured with CSF-1 and ILs released from AC10 hydrogels. Indeed, the typical reparative heterogeneous population, composed of round macrophages (identified with white arrowheads in Figure 3B) and cells presenting cytoplasmic extensions with an elongated cell body (identified with red arrows in Figure 3B; Ploeger et al., 2013) was widely represented in presence of ILs. Antibodies against CD206 and CD163 were used to assess polarization of monocytes toward reparative phenotypes (Yang et al., 2014; Kumar et al., 2016). Similarly to what was observed with the morphological assessment, confocal imaging confirmed that the percentage of cells positive for M2 surface markers was significantly increased in the presence of medium containing the differentiation factors released from the hydrogels in comparison with the control, represented by untreated monocytes (*p* < 0.05, Figures 3C,D). Monocytes exposed to extracts of AC10 hydrogels loaded with combinations of CSF-1 and IL4 exhibited a slight but not statistically significant increase in the level of CD163^+^ macrophages, compared to CSF-1 only (*p* ≥ 0.05), and a marked increase of CD206^+^ macrophage subtypes (^∗∗^*p* < 0.01) (Figures 3C,D). As expected, the addition of IL6 and IL13 cytokines induced both a strong CD163 and CD206 up-regulation at day 10 compared to the other test groups (^∗∗∗^*p* < 0.0001, Figures 3C,D). Overall, these data indicate that the immobilization and release of immunomodulatory cytokines from alginate hydrogels did not impair their biological activity, resulting in an efficient polarization of monocytes, comparable to the stimulation with fresh cytokines stock solutions added to the culture medium (Supplementary Figure S2).

### Wound Healing in the Rat Ischemic Skin Flap Model Is Enhanced With Delivery of CSF-1 and IL4

**FIGURE 4:**
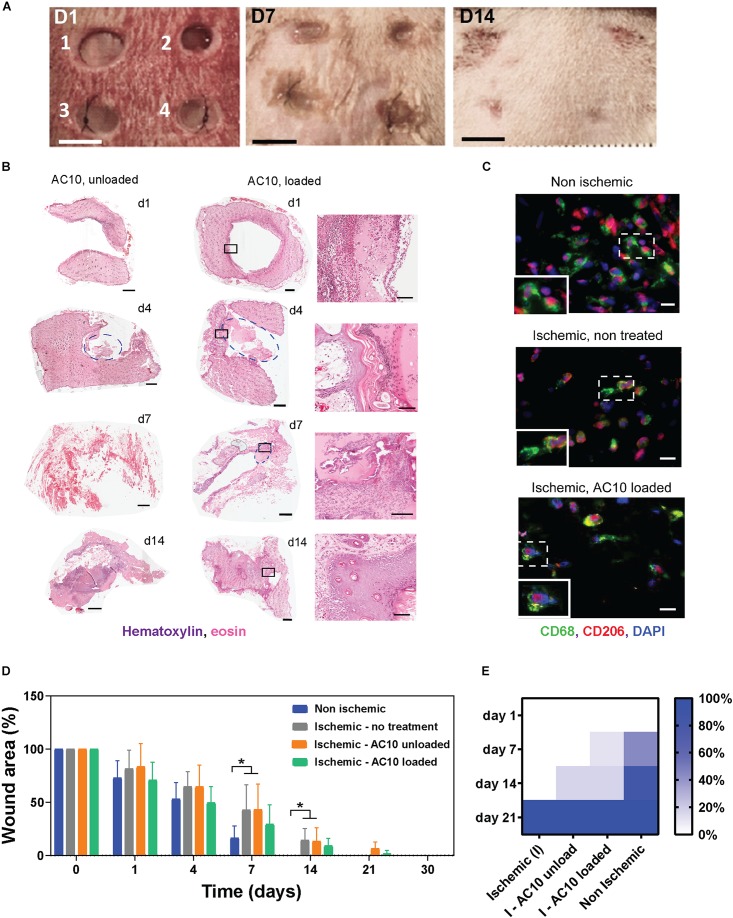
The efficacy of the immunomodulatory biomaterials is tested in a predictive ischemic skin flap model. **(A)** Digital images of ischemic wounds at 0, 3, 7, and 14 days. Wounds received (1 and 2) no hydrogels, (3) unloaded alginate hydrogels, or (4) alginate hydrogels loaded with 2 μg CSF-1 and 2 μg IL4. Tissue necrosis is visible at day 14 in wounds 1 and 2. Scale bar = 5 mm. **(B)** Representative hematoxylin/eosin staining of wound tissues treated with AC10 hydrogels unloaded or loaded with 2 μg CSF-1 and 2 μg IL4 at 1, 4, 7, and 14 days post ischemia (scale bar = 1 mm). Hydrogels are identified with a blue circle. Some of the hydrogels detached from the slides during the histological processing (for example, wound image at day 1). The black squares on the H&E staining of loaded AC10 hydrogels indicate the area represented at higher magnification on the right of each sample (scale bar = 200 μm. **(C)** Immune cells stained with CD68 (pan macrophage marker) and CD206 (reparative macrophage phenotypes) at the wound edges at 4 days post ischemia, scale bars = 20 μm. A 30 μm × 45 μm magnified area is shown in the bottom-left inset of each image. **(D)** Kinetics of wound closure for the four experimental conditions show increased wound healing in presence of cytokines. *N* = 4–9 wounds were analyzed at different time points for each group. **(E)** Fraction of healed wounds for the tested groups at different time points (I = ischemic, NI = non-ischemic). At 21 days, all wounds were completely healed.

To evaluate the reparative potential of the combination of CSF-1 and IL4 released from the biomaterials in ischemic tissues, we have adapted a predictive model of ischemic skin wounds created with a bipedicle skin flap (Figure 4A). With this model, a silicon membrane is placed beneath the flap as an inert barrier to inhibit vascularization of the skin, thus creating an ischemic site. Four wounds were created in the ischemic skin, and the alginate hydrogels releasing immunomodulatory cytokines were implanted. Evaluation of digital images and histological sections of the skin near the epithelial gap by hematoxylin/eosin staining allowed measurement of the kinetics of wound healing and hydrogel degradation, which was typically observed at 3–5 days post ischemia (Figure 4B). Phenotypic evaluation of the immune cell population was conducted by immunohistochemistry with CD68 (pan-macrophages marker) and CD206 (reparative macrophages marker) antibodies at 4 days post ischemia. Results show little or no differences among groups. The extensive co-localization of CD68 and CD206 markers, which can be associated with the presence of reparative macrophages subsets in the ischemic and non-ischemic wounds, indicates initiation of the healing process via a shifted macrophage polarization (Figure 4C). The limited presence of CD68 + CD80 + cells, associated with inflammatory macrophages phenotypes, at 4 days corroborates with the evidence of a rapid onset of the reparative process (Supplementary Figure S3).

A significant delay in wound healing was observed for the ischemic wounds compared to the non-ischemic controls, as shown in Figure 4D (*p* < 0.05 at days 7 and 14 for ischemic wounds that received no hydrogels or unloaded AC10 compared to non-ischemic control wounds). Importantly, accelerated healing trends were observed with delivery of CSF-1 and IL4 (2 μg each) from AC10 alginate hydrogels, compared to sham control and to wounds treated with unloaded alginate hydrogels (Figures 4D,E). Faster wound healing is obtained with the new therapeutic treatment compared to the unloaded controls (12% increase in the healed wound area at day 14). A chi-squared test performed on the fraction of healed wounds at 1, 7, 14, and 21 days (Figure 4E) showed statistical differences in the healing kinetics between cytokine-loaded AC10 hydrogels compared to the untreated ischemic wound (*p* < 0.001) and the unloaded AC10 hydrogels (*p* < 0.05).

### Injection of Immunomodulatory Biomaterials in the Infarcted Heart Induce Recovery of Cardiac Function at 15 Days Post Myocardial Infarction

**FIGURE 5:**
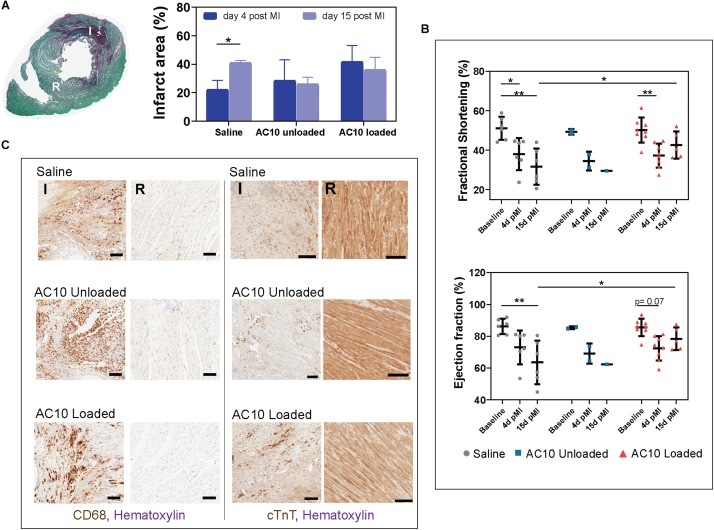
Infarcted rat hearts receiving the immunomodulatory therapy show improvement in cardiac function and polarization of macrophages in the ischemic wound. **(A)** Cross section of a heart labeled with picrosirius red/fast green allow identification of the infarct area in red (I) and of the remote healthy tissue (R). Quantification of infarct area relative to the left ventricle area for all groups is shown on the right. Data are represented as mean ± standard deviation. *N* = 3 hearts were evaluated for all groups except AC10 unloaded- 15 days (*n* = 2) and AC10 loaded- 15 days (*n* = 10). **(B)** Cardiac function evaluated by fractional shortening (%) and ejection fraction (%) over 15 days. Each symbol represents the fractional shortening or ejection fraction of an individual rat. Data are expressed as mean ± standard deviation. **(C)** CD68 and cTnT staining for assessing macrophages and cardiomyocyte morphology in the infarct and remote area of the heart at day 15, scale bars = 100 μm.

**FIGURE 6:**
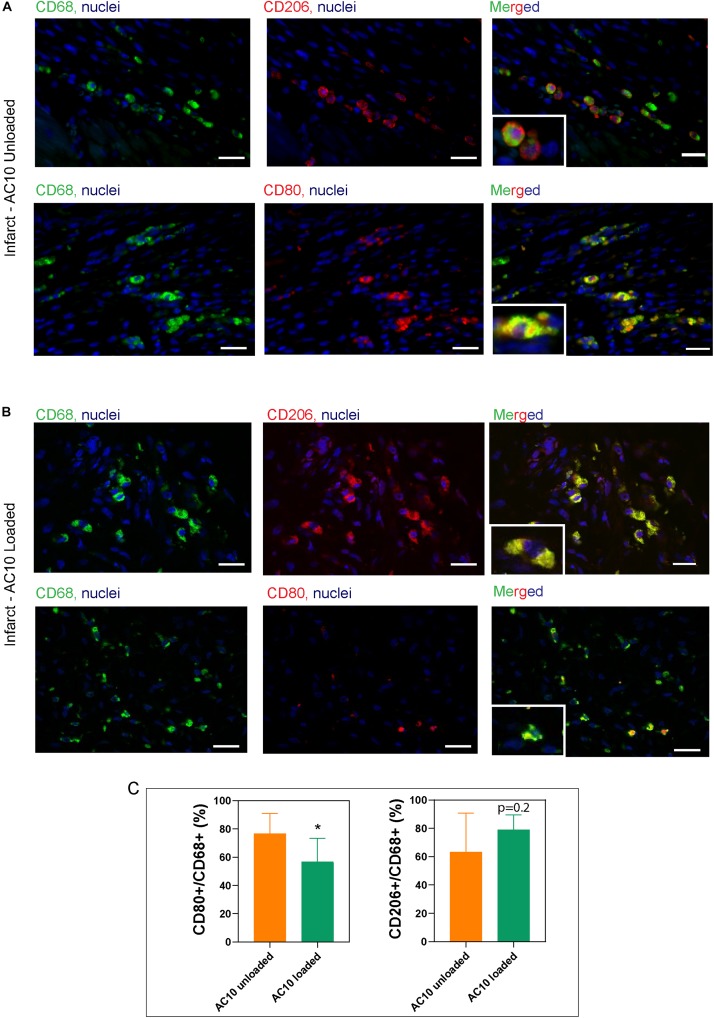
Identification of macrophage subtypes in the infarct area shows the presence of reparative phenotypes in hearts treated with CSF-1 and IL4 loaded alginate hydrogels. Representative images of the infarct scar treated with unloaded AC10 hydrogels **(A)** or cytokine-loaded AC10 hydrogels **(B)** show macrophages stained with antibodies against CD68 (pan macrophage marker: left column), CD80 (inflammatory subsets: top row of A,B) and CD206 (reparative subsets: bottom row of A,B), scale bars = 20 μm. A 25 μm × 35 μm magnified area is shown in the bottom-left inset of each image. **(C)** Quantification for the macrophage phenotypes normalized to CD68 + cells was performed on *n* = 198 ± 47 CD68 + cells per group (*p < 0.05).

Injectable formulations of AC10 hydrogels were introduced into the infarcted myocardium of wild type rats to evaluate their regenerative potential. Fully immunocompetent rats received MI by 60 min of ischemia then reperfusion, followed by intramyocardial injection of either (Wilkins et al., 2017) saline, (Benjamin et al., 2019) unloaded AC10, or (Shimokawa and Yasuda, 2008) AC10 loaded with CSF-1 and IL4 (2 μg each). Measurements of the infarct area on picrosirius red/fast green stained heart sections showed no difference in the size of the infarcts for the three groups at 4 and 15 days post MI; however, a significant increase in scar size from 4 to 15 days was visible for the group treated with saline injections (Figure 5A). Results from echocardiographic examination showed a decrease in cardiac function (measured as a 29.6 ± 1.0% decrease in fractional shortening and 18.3 ± 0.7% decrease in ejection fraction, averaging all groups) at 4 days post MI compared to the baseline (Figure 5B). A higher fractional shortening at day 15 between the group treated with the biomaterials loaded with the immunomodulatory cues versus sham treatment suggests the therapy improves functional outcomes (Figure 5B). Histological assessment with CD68, pan macrophage marker, and cardiac Troponin T (cTnT), cardiac marker, shows a broad presence of macrophages in the infarct area with few surviving cardiomyocytes (Figure 5C). The presence of inflammatory versus reparative macrophages was evaluated in the infarct area by use of CD68, CD206 (reparative macrophage phenotypes), and CD80 (inflammatory macrophages phenotypes). CD206

expression is specific for the M2a macrophage sub-phenotype, that is known to be induced *in vitro* by IL-4 or IL-13 (Ma et al., 2018), and is therefore the marker selected for assessing the healing-type macrophages stimulated *in vivo* with our therapeutic. The significant decrease of CD80 positive macrophages in the infarct area of rat hearts receiving AC10 loaded hydrogels compared to the other treatment groups suggest possible transition away from inflammation and toward healing/repair (Figure 6). Presence of CD68 + CD206 + healing macrophages appears elevated in the infarct region of animals treated with either unloaded and cytokines-loaded injectable biomaterials, but with no statistical difference among groups, likely due to the high biological variability between animals.

## Discussion

In this study, we evaluate an innovative approach to address the need of an effective treatment for repairing damaged myocardium after an infarction, which uses biomaterials for the localized release of immunomodulatory cytokines. Previous works have explored immunomodulatory strategies for treating the infarcted myocardium, primarily by preventing excessive inflammation with anti-inflammatory or immunosuppressive agents targeting select pathways, initiators of inflammation (such as reactive oxygen species), inflammatory cytokines or immune cells, including B and T lymphocytes (Panahi et al., 2018). Here, we use CSF-1 and anti-inflammatory interleukins IL4, IL6, and IL13 to target monocytes and macrophages for initiating healing, repair and revascularization processes in the ischemic myocardium. In corroboration with results obtained *in vitro* in previous studies (Rovida et al., 2001; Mia et al., 2014; Boulakirba et al., 2018; Holloway, 2018), we were able to obtain a phenotypic switch of human blood monocytes toward pro-healing macrophage subtypes by treating cells with a combination of CSF-1 and IL4. Further, we have demonstrated that the efficacy of polarization increases with the use of a cocktail of anti-inflammatory interleukins, including IL6 and IL13 *in vitro*. Previous works in the literature show that CSF-1 is required for enhancing the final activation status of reparative macrophage phenotypes when cells are treated with a cocktail of anti-inflammatory cytokines (Mia et al., 2014; Zarif et al., 2016). Based on this evidence, we selected a combination of CSF-1 and IL4 for *in vivo* assessment as the most simple delivery system that elicits polarization of monocytes toward reparative macrophage sub-populations (Figure 3).

We used this acellular biomaterials system for the minimally invasive treatment and the localized delivery of “repair-instructive” cytokines in the setting of ischemic skin tissue and acute MI.

Soft alginate biomaterials are specifically designed (1) to gel at 37°C in 5–10 min, being optimal carriers for *in situ* gel formation; (2) to optimize timing and location of the treatment, tailoring their release to target the circulating monocytes and macrophages during the acute infarct phase (0–4 days) and prior to the formation of granulation tissue (4–14 days) (Ferrini et al., 2019); and (3) to rapidly biodegrade *in vivo* after releasing the therapeutics to avoid biomaterial-induced arrhythmias (Hernandez and Christman, 2017; Figures 1, 2).

The efficacy of the developed immunomodulatory therapeutic has been evaluated *in vitro* (Figure 3) and in the ischemic skin flap model, a reliable model to study the effects of tissue ischemia on wound healing (Figure 4). This *in vivo* model recapitulates the pathology of ischemic wounds, with impaired vascularization and delayed wound healing (Schwarz et al., 1995; Gould et al., 2005; Trujillo et al., 2015; Patil et al., 2017), offering a convenient predictive model to optimize the immunomodulatory biomaterials prior to further testing in the infarcted myocardium. By measuring the epithelial gap (the distance between the wound margins) up to 21 days in ischemic and non-ischemic wounds, we demonstrate an accelerated healing of the ischemic wounds treated with the immunomodulatory biomaterials. Indeed, ischemic wound repair with CSF-1 and IL4 delivery was different from the non-treated ischemic wound and the unloaded control healing kinetics (Figure 4E). The promising healing trends obtained in this work with the delivery of immunomodulatory cytokines in ischemic rat skin wounds (Figure 4) show similar regenerative potential with the results of previous studies investigating topical applications or subcutaneous injections of exogenous pro-angiogenic growth factors, including vascular endothelial growth factor (VEGF) (Zhang et al., 2003), fibroblast growth factor (FGF) (Fayazzadeh et al., 2016) and platelet-derived growth factor (PDGF) (Chin et al., 2002). The beneficial effects of the immunomodulatory treatment observed in the skin flap model of ischemia provided the rationale for evaluating the immunomodulatory treatment in the MI, a more complex, non-healing wound generated by ischemia in the heart. We performed MI by ischemia/reperfusion injury, which is an established model to assess cardiac regeneration after MI (Lindsey et al., 2018). Compared to a permanent ligation procedure, the I/R model is more clinically-relevant, as it recapitulates reperfusion injury as observed in human patients, thus enabling a more appropriate study of the immune system response in an animal model due to the onset of inflammation (Chimenti et al., 2004).

Cardiomyocyte death following ischemia activates the innate immune response, and monocytes and inflammatory macrophages are rapidly recruited into the infarcted wound bed to remove dead cells and tissue debris. However, if a rapid polarization toward reparative macrophage phenotypes is not achieved within a few days, the heart progresses rapidly through left ventricular remodeling, accompanied by deposition of collagenous scar and more severe functional decline, that can lead to heart failure (Ferrini et al., 2019).

Our therapeutic intervention aims to leverage the native immune cell population in the infarct during the acute inflammatory phase to alter the course of remodeling through pro-angiogenic processes. The soft alginate hydrogels produced in this study are designed as injectable carriers for the immunomodulatory cytokines and are not aimed at providing mechanical support to the infarcted area. The rheological properties of AC10 hydrogels are appropriate for intramyocardial injection via small needles (30G), and the viscoelastic behavior of AC10 hydrogels is comparable to that of other injectable biomaterials described in the literature (Oliveira et al., 2010; Munarin et al., 2014). Other acellular biomaterials have been injected in the myocardium with minimally invasive procedures for biomaterial-alone approaches or using biomaterials as carriers of growth factors, microribonucleic acids (miRNAs) or exosomes (Hernandez and Christman, 2017). However, to the best of our knowledge, this is the first study that reports the injection of biomaterials loaded with a cocktail of CSF-1 and IL4 as immunomodulatory cues for improving cardiac repair. Our study suggests that the cytokine-loaded hydrogels induce changes of the immune cell population in the infarct environment that reduces inflammatory phenotypes to tip the balance toward that results in improved heart function at 15 days post-MI. These results warrant further investigation of this injectable therapeutic system for advancing the healing process in the acute phase potentially via macrophage-driven revascularization that may reduce chronic ischemia for surviving cardiomyocytes and support subsequent long-term treatments of the infarct.

## Data Availability Statement

The datasets generated for this study are available on request to the corresponding author.

## Ethics Statement

The animal study was reviewed and approved by Brown University and Rhode Island Hospital Institutional Animal Care and Use Committee (IACUC protocol No. 1702000256).

## Author Contributions

FM and KC developed the idea and edited the manuscript FM, KC, NB, and LV designed and directed the experiments and interpreted the data. FM and IR optimized the preparation of alginate hydrogels and performed rheological analysis and stability studies. NB performed *in vitro* experiments to assess polarization of monocytes and release kinetics from alginate hydrogels. GM contributed in monocytes isolation and characterization. CP, FM, and IR were involved with surgical procedures and post-operative animal care, histological preparations, and data analysis. FM supervised the work. NB, IR, and FM wrote the manuscript with critical inputs from all the authors.

## Conflict of Interest

The authors declare that the research was conducted in the absence of any commercial or financial relationships that could be construed as a potential conflict of interest.
