# Supplementary material for: Engineering Immunomodulatory Biomaterials for Regenerating the Infarcted Myocardium
Source: Front Bioeng Biotechnol. 2020 Apr 7;8:292. doi: 10.3389/fbioe.2020.00292 (PMC7154131; doi:10.3389/fbioe.2020.00292)
Supplement: Supplementary file 1 [file Data_Sheet_1.docx]

**Supplemental Methods.**

# Histology and Immunohistochemistry protocols

## Immunohistochemistry of monocyte cultures

Monocytes cultured with the immunomodulatory treatment were washed with 1X PBS, fixed with 4% (w/v) paraformaldehyde solution (PF) for 20 min at 4°C and after washing, blocked with PBS containing 1% (w/v) bovine serum albumin for 1 h at room temperature (RT). Cells were then incubated with rabbit primary antibody against human CD206 (1:100 dilution, OriGene Technologies, Maryland, USA) overnight at 4°C. Subsequently, coincubation of mouse anti-humanCD163-PE (1:200 dilution, OriGene Technologies), and anti-rabbit-Alexa 633 conjugated antibodies (1:250 dilution, Invitrogen) was performed for 1 h at RT. After 1X PBS washing, cells were counterstained for 5 min with a solution of Hoechst 33342 (2 μg/mL) to target nuclei.

## Histology and immunohistochemistry of skin wound tissue and infarcted myocardium

PF-fixed tissue sections were de-paraffinized with xylenes and re-hydrated with decreasing concentrations of ethanol, followed by washes in 1x PBS. For picrosirius red/fast green staining, slides were put in picrosirius red/fast green solution (0.1% Sirius Red and 0.1% Fast Green in Picric Acid) for 30 minutes, were quickly washed with water and de-hydrated with ethanol. Slides were then put in xylenes (2x 5 min) and were coversilpped with Permount (Electron Microscopy Science) mounting medium. For hematoxyilin/eosin staining, re-hydrated slides were stained with filtered hematoxylin (4 minutes) followed by quick rinse in H_2_O, differentiation in 1% acid alcohol, rinse in H_2_O and Scott’s Blue (5 minutes) before de-hydration with ethanol and coverslipping. For CD68 and cTnT staining with DAB development, peroxidase quenching was obtained with 1:10 H_2_O_2_ in cold methanol for at least 15 minutes during re-hydration, followed by antigen retrieval by incubation in boiling citrate buffer (10 minutes). Sections were allowed to cool for 20 minutes in the citrate buffer and were subsequently blocked with 1.5% normal goat serum in ICC PBS (1h) and incubated in 1:100 mouse anti rat CD68 (MCA341B, Bio-rad) or 1:100 mouse anti-cTnT (MS-295-P1, ThermoFisher Scientific) overnight. Antibody detection was performed with biotin-SP goat antimouse IgG (H+L) (1:500) (115-065-003, Jackson Immunoresearch) followed by ABC (Vector Labs) and DAB (Vector Labs) development. Nuclei counterstaining was obtained with hematoxylin. For macrophage phenotype immunohistochemistry, sections were re-hydrated, then antigen retrieval in citrate buffer and blocking steps were performed as described above. Double staining was achieved by incubation with mouse anti-CD68 antibody (ab31630, Abcam) and rabbit Anti-Mannose Receptor antibody (Anti-CD206) (ab64693, Abcam) or mouse anti-CD68 antibody (ab31630, Abcam) and rabbit Anti-CD80 antibody (ab134120, Abcam) overnight followed by Alexafluor 488-conjugated goat anti-mouse (Invitrogen) and Alexafluor 594-conjugated goat anti-rabbit (Invitrogen) for 1h. Sections were mounted with VECTASHIELD® with DAPI (Vector Labs) for nuclei detection.
