# Supplementary material for: Engineering Immunomodulatory Biomaterials for Regenerating the Infarcted Myocardium
Source: Front Bioeng Biotechnol. 2020 Apr 7;8:292. doi: 10.3389/fbioe.2020.00292 (PMC7154131; doi:10.3389/fbioe.2020.00292)
Supplement: Supplementary file 2 [file Table_1.DOCX]

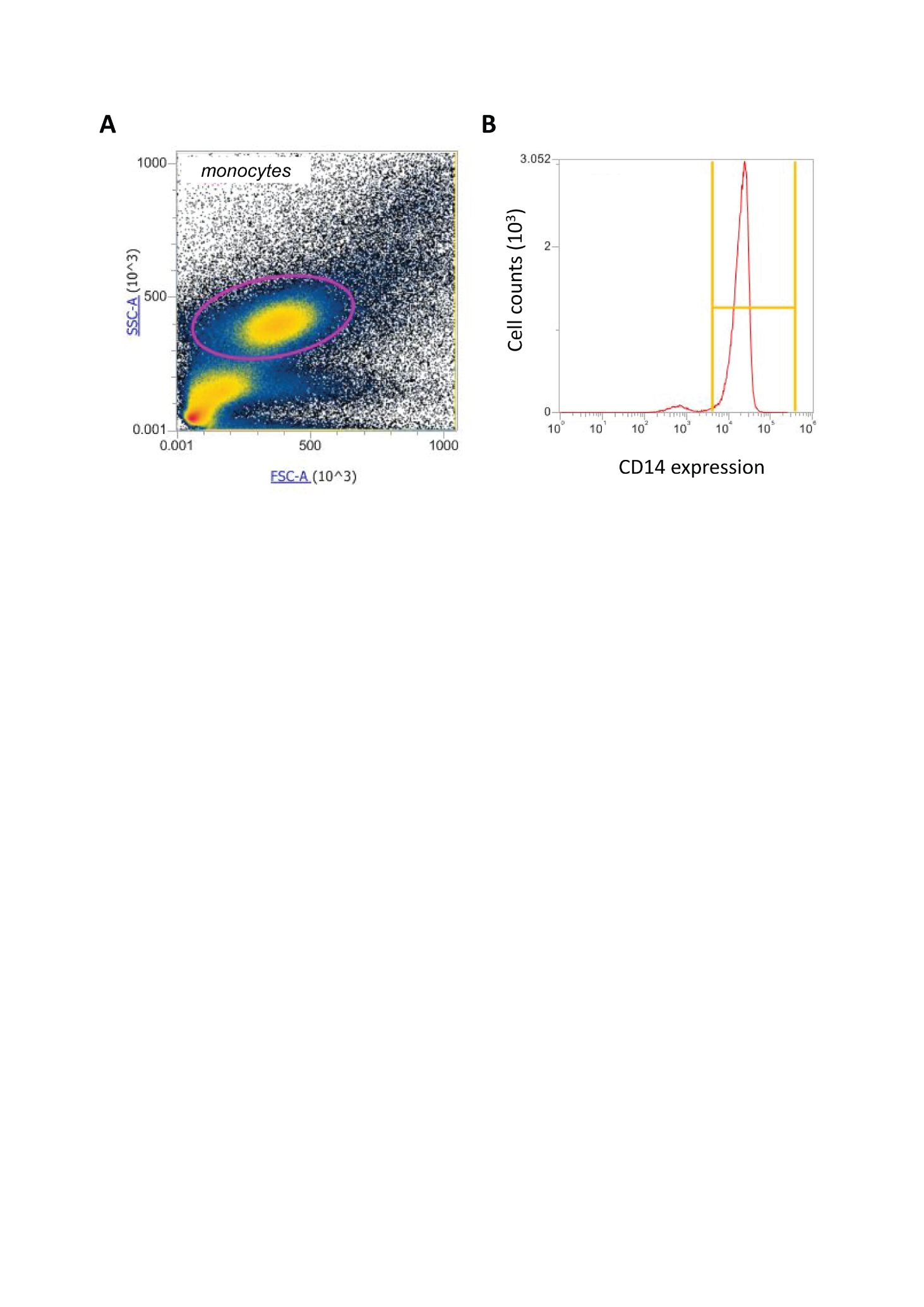


*Supplemental Figure S1. FACS analysis of monocytes isolated from human peripheral blood samples shows 85% CD14+.* Representative forward/side scatter plot (**A**) and CD14 expression graph (**B**) from a single donor sample.
