# Supplementary material for: Engineering Immunomodulatory Biomaterials for Regenerating the Infarcted Myocardium
Source: Front Bioeng Biotechnol. 2020 Apr 7;8:292. doi: 10.3389/fbioe.2020.00292 (PMC7154131; doi:10.3389/fbioe.2020.00292)
Supplement: Supplementary file 3 [file Table_2.doc]

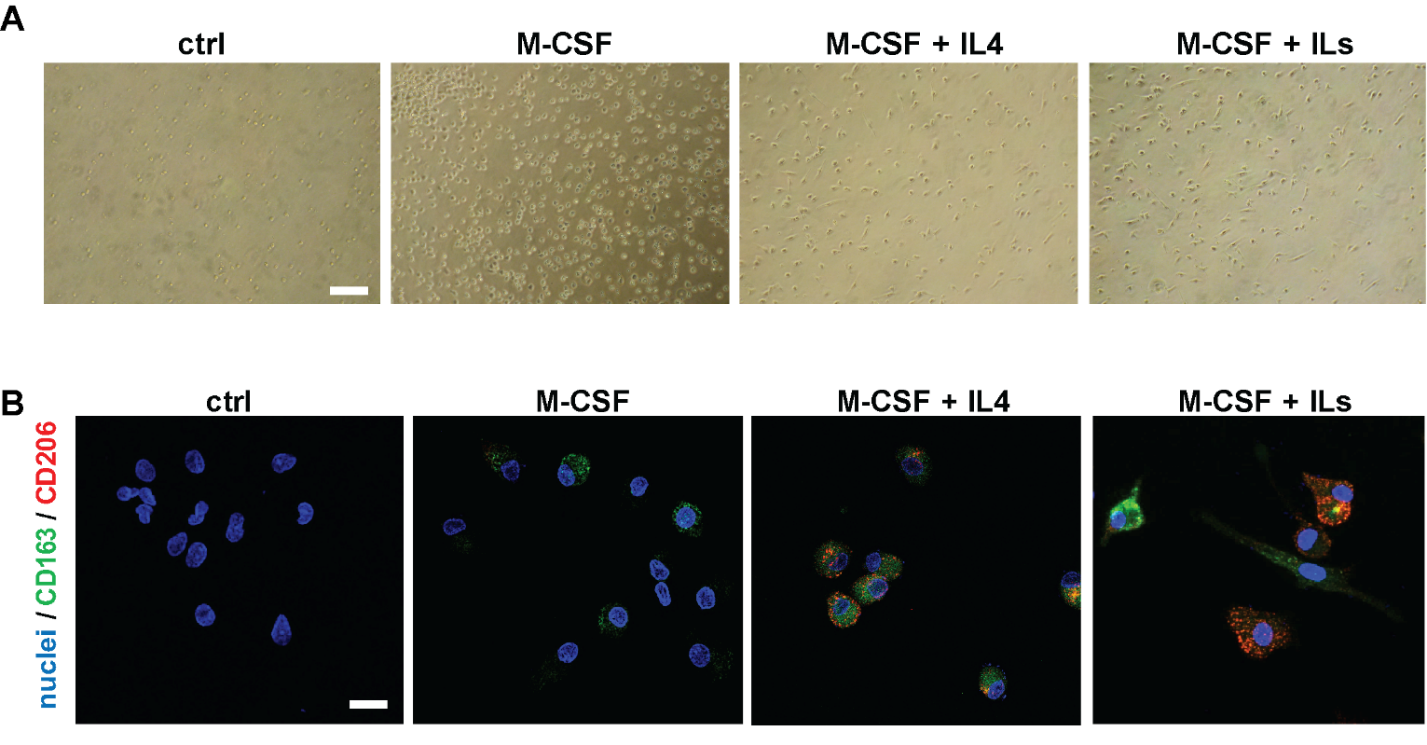


*Supplemental Figure S2. Polarization of monocytes towards reparative macrophage subpopulations is obtained adding immunomodulatory cytokines (CSF-1, IL4/6/13, 20 ng/mL) to the culture medium*. Assessment of (**A**) morphology by optical light microcopy and (**B**) expression of typical M2 macrophages markers (CD163, green and CD206, red) by confocal laser scanning microscopy after 10 days of culture. Nuclei were counterstained with Hoechst 33342 (blue). Scale bars: 100 μm (A) and 20 μm (B), respectively.
