# Supplementary material for: Engineering Immunomodulatory Biomaterials for Regenerating the Infarcted Myocardium
Source: Front Bioeng Biotechnol. 2020 Apr 7;8:292. doi: 10.3389/fbioe.2020.00292 (PMC7154131; doi:10.3389/fbioe.2020.00292)
Supplement: Supplementary file 4 [file Table_3.DOCX]

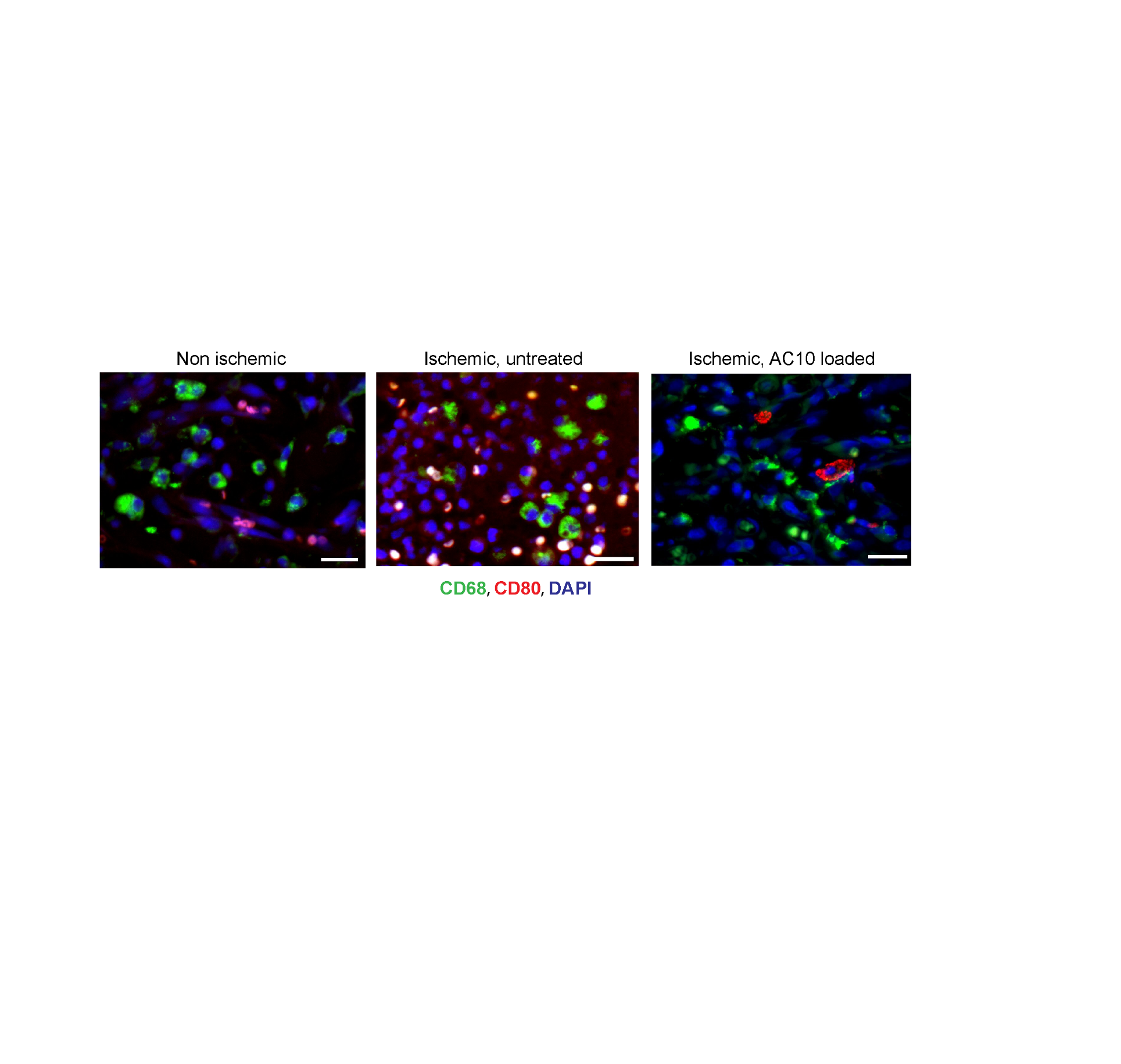


*Supplemental Figure S3. Immune cells stained with CD68 (pan macrophage marker) and CD80 (inflammatory macrophage phenotype) at the wound edges at 4 days post ischemia, scale bars= 20 µm.*
